# MicroRNA-296-5p inhibits cell metastasis and invasion in nasopharyngeal carcinoma by reversing transforming growth factor-β-induced epithelial–mesenchymal transition

**DOI:** 10.1186/s11658-020-00240-x

**Published:** 2020-11-03

**Authors:** Meihui Chen, Chen Chen, Haiqing Luo, Jing Ren, Qiuqin Dai, Wenjia Hu, Keyuan Zhou, Xudong Tang, Xiangyong Li

**Affiliations:** 1 Institute of Biochemistry and Molecular Biology of Guangdong Medical University, No. 2 Wenming Dong Road, Xiashan District, Zhanjiang, 524023 Guangdong China; 2 Department of Clinical Laboratory of Zhanjiang Central Hospital, Zhanjiang, 524023 China; 3 Center of Oncology of The Affiliated Hospital of Guangdong Medical University, Zhanjiang, 524023 China

**Keywords:** miR-296-5p, Metastasis, Invasion, EMT, Nasopharyngeal carcinoma

## Abstract

**Aim:**

To explore the effect of miR-296-5p on the metastasis of nasopharyngeal carcinoma (NPC) cells and investigate the underlying mechanism.

**Methods:**

The expressions of miR-296-5p in NPC tissues and cells were determined using GSE32920 database analysis and real-time PCR and miRNA microarray assays. An miR-296-5p mimic and inhibitor were transfected into NPC cells. Then, immunofluorescence imaging, scratch wound-healing, transwell migration and invasion assays were used to observe the effects of miR-296-5p on cell metastasis and invasion. Real-time PCR and western blotting were carried out to detect the expressions of genes and proteins related to epithelial–mesenchymal transition (EMT). A dual luciferase reporter assay was used to identify whether TGF-β is the target gene of miR-296-5p. Finally, TGF-β expression plasmids were transfected into NPC cells to verify the role of TGF-β in the miR-296-5p-mediated inhibition of nasopharyngeal carcinoma cell metastasis.

**Results:**

Our results show that miR-296-5p inhibits the migratory and invasive capacities of NPC cells by targeting TGF-β, which suppresses EMT. Importantly, the miR-296-5p level was significantly lower in human NPC tissues than in adjacent normal tissues. It also negatively correlated with TGF-β and was significantly associated with the lymph node metastasis of patients with NPC.

**Conclusions:**

Our findings show that miR-296-5p represses the EMT-related metastasis of NPC by targeting TGF-β. This provides new insight into the role of miR-296-5p in regulating NPC metastasis and invasiveness.

## Background

Nasopharyngeal carcinoma (NPC) is a major cause of cancer-related mortality in Southern Asia, especially in southern China, where the annual incidence is as high as 35–50 cases per 100,000 people [1, 2]. The 5-year overall survival rate has obviously increased thanks to the clinical implementation of numerous new therapeutic strategies, including intensity-modulated radiation therapy [3, 4]. Despite this, NPC patients with recurrence and metastasis post-radiation or radiochemotherapy still face a dismal survival rate. Thus, the mechanisms underlying the progression and metastasis of NPC need to be explored.

Epithelial–mesenchymal transition (EMT) is a prerequisite for tumor invasion and metastasis as it causes the necessary loss of cellular polarity and adhesion [5, 6]. Several signaling pathways have been implicated in the regulation of EMT, particularly the transforming growth factor-β/Smad (TGF-β/Smad) pathways [7, 8]. TGF-β is a multifunctional regulatory protein that can regulate multiple cellular processes, and is known to be particularly important in tumor metastasis [9, 10]. It promotes tumor progression by facilitating the invasion of various types of tumor cells via EMT [11, 12]. The underlying regulatory mechanisms of TGF-β-induced EMT in NPC remain unclear.

MicroRNAs (miRNAs, miRs) are endogenously expressed small non-coding RNAs that generally regulate gene expression by binding to the 3ʹ untranslated region (3ʹ-UTR) of their targets, resulting in silencing [13]. Numerous studies have shown that they play crucial roles in human cancer development, with particular impact on cancer cell proliferation, differentiation, cell cycle and apoptosis [14–16]. Thus, they’re involved in regulating the progression and metastasis of tumors [17–19]. Aberrant expression of several miRNAs, notably miRNA-296-5p, has been broadly implicated in the pathogenesis of NPC [9, 20]. Therefore, their dysregulation may participate in NPC cell invasion and migration, contributing to its progression and metastasis.

In this study, we established that miR-296-5p has a significantly lower expression in NPC tissues and cells and was involved in regulating the EMT and metastasis of NPC cells in vitro. In addition, we demonstrated for the first time that miR-296-5p directly targets TGF-β and reverses the TGF-β-induced EMT of NPC. This study revealed a novel mechanism by which miR-296-5p regulates the EMT and metastasis of NPC cells, indicating that it could be a new target for the treatment of NPC.

## Methods

### Clinical specimens

Paired NPC tissue and adjacent normal tissue samples were obtained from 15 confirmed NPC patients via fiber optic nasopharyngoscopy. The procedure was performed at the Affiliated Hospital of Guangdong Medical University. The Ethics Committee of the Affiliated Hospital of Guangdong Medical University approved the protocol for the clinical study.

### Cell culture and transfection

The cell culture was carried out as described in our previous publication [16]. Briefly, NPC cell lines CNE-1, CNE-2, C666-1, 6-10B, 5-8F and HONE1 were maintained in RPMI 1640 media with 10% FBS, 100 U/ml penicillin and 100 mg/ml streptomycin at 37 ℃ in a humidified incubator with 5% CO_2_. In addition, NP69, an immortalized nasopharyngeal epithelium cell line, was cultured in the defined keratinocyte serum-free medium (Life Technologies) with specific growth factors.

The miR-296-5p mimic, miR-296-5p inhibitor, TGF-β expression plasmids and corresponding control vectors were transfected into the NPC cells with Lipofectamine 3000 reagent as per the manufacturers’ protocols.

### Scratch wound-healing assay

The migratory capacities of the transfected NPC cells were assessed using the wound-healing assay as described previously [19]. Briefly, NPC cells were cultured in 6-well plates to 90% confluence after transfection. Artificial parallel scratches were made with pipette tubes. After the plates were washed with PBS, the cells were cultured in an incubator. Finally, we photographed the cell wound healing at different time points with a microscope.

### Transwell assays

A 24-well transwell chamber was used to detect the migratory and invasive abilities of NPC cells according to the manufacturer’s protocols. Briefly, after 48 h of culture, 1 × 10^5^ cells from each treatment group were seeded into the upper chamber in 200 µl medium with serum and Matrigel (1:8 dilution) for the invasion assay. Subsequently, 500 µl medium with 20% FBS was added into the lower chamber. After 24 h of culture, the cells were fixed using 4% paraformaldehyde and then stained using crystal violet. Finally, five random fields of cells were counted in each well through an optical microscope.

### Immunofluorescence imaging

A FITC-Phalloidin staining assay was performed to investigate the influence of miR-296-5p and TGF-β on the cytoskeletal structure of NPC cells. Briefly, after round cover glasses were placed into 12-well plates, 1.8 × 10^4^ cells from each treatment group were seeded and cultured for 24 h. Then the cover glasses were fixed in 4% paraformaldehyde for 15 min and permeabilized with 0.1% Triton X-100 for 20 min. Finally, cells were continuously incubated with 200 μl of FITC-labeled phalloidin (200 U/ml, KeyGen BioTECH) containing 1% BSA for 30 min in the dark at room temperature, and were stained with 100 μl of DAPI solution (Beyotime). An Olympus microscope (Olympus, Japan) was used to image the transfected cells.

### Real-time PCR

Real-time PCR were carried out as described in our previous paper [16]. Briefly, total RNA was extracted using an miRcute miRNA isolation kit (TIANGEN) for miRNA and TRIzol (Life Technologies) for mRNA in accordance with the manufacturer’s instructions. Then, miRNA and mRNA were polyadenylated using a poly-A polymerase-based First-Strand Synthesis kit (Takara). Subsequently, a PrimeScript RT Reagent kit (TaKaRa) were used for reverse transcription (RT) of the total mRNA. Finally, miR-296-5p and complementary DNA (cDNA) in these samples were assayed using SYBR Green I (Applied Biosystems). The primers were: CDH2 (forwards: 5′-ATCCCACATATGGCCTTTCA-3′ and reverse: 5ʹ-ATCCCACATATGGCCTTTCA-3ʹ), CTNNB1 (forwards: 5′-AAGTGCCTGACACACTAACCAA-3′ and reverse: 5′-CAAGCAAGGCTAGGGTTTGA-3′), ZEB1 (forwards: 5′-CCTTAATCCTCCGCATTTCA-3′ and reverse: 5′-CC CTGTTAGGCAGTGAGGAA-3′), SNAIL1 (forwards: 5′-CATGGCCATTTCTGTGGAG-3′ and reverse: 5′-GGAGCTTCCCAGTGAGTCTG-3′), TWIST (forwards: 5′-GTCCGCAGTCTTACGAGGAG-3′ and reverse: 5′-CCAGCTTGAGGGTCTGAATC-3′), MMP2 (forwards: 5′-TTGACGGTAAGGACGGACTC-3′ and reverse: 5′-ACTTGCAGTACTCCCCATCG-3′), MMP9 (forwards: 5′-TGGGAAGTACTGGCGATTCT-3′ and reverse: 5′-CCTGTGTACACCCACACCTG-3′), SMAD2 (forwards: 5′-AGCAGAATACCGAAGGCAGA-3′ and reverse: 5′-TCATGGGACTTGATTGGTGA-3′), SMAD3 (forwards: 5′-CCCCAGAGCAATATTCCAGA-3′ and reverse: 5′-GGCTCGCAGTAGGTAACTGG-3′), SMAD4 (forwards: 5′-TGCATGACTTTGAGGGACAG-3′ and reverse: 5′-GTGGAA GCCACAGGAATGTT-3′).

### Western blotting

Western blotting was carried out as described previously [16]. First, protein extracts were separated via electrophoresis on 10% SDS polyacrylamide gel and transferred onto PVDF membranes (Millipore). Then, these membranes were incubated at 4 °C overnight with the primary antibody, washed 3–5 times with, and incubated with the corresponding secondary antibodies for 1 h at room temperature. Finally, chemiluminescent detection was performed using enhanced chemiluminescence (Cell Signaling Technology).

### Dual luciferase reporter assay

The target gene of miR-296-5p was identified using a dual luciferase reporter assay. First, the pmiR-RB-Report vector with wild-type 3ʹ-UTRs of TGF-β (wt) or mutant TGF-β 3ʹ-UTRs (mut) were obtained from RiboBio (China). Then, wt or mut reporter constructs and miR-296-5p mimics or inhibitors were co-transfected into NPC cells with Lipofectamine 3000 (Invitrogen). After 48 h of incubation, passive lysis buffer was use to lyse the cells, which were further assayed using the Dual Luciferase Reporter Assay kit (Promega, USA) according to the manufacturer’s protocol. Renilla luciferase assay was used to normalize the luciferase activity of each lysate.

### Statistical analysis

SPSS 19.0 software (IBM Inc., USA) was used for the statistical analyses in this study. The results are presented as means ± SD based on 3 independent experiments. The differences between two groups were evaluated using Student’s t test. The correlation between miR-296-5p and targeted gene expressions in NPC tissues was evaluated with Pearson’s correlation tests. When p < 0.05, the difference was considered statistically significant.

## Results

### MiR-296-5p exhibited reduced expression in NPC tissues and cell lines

**Fig. 1:**
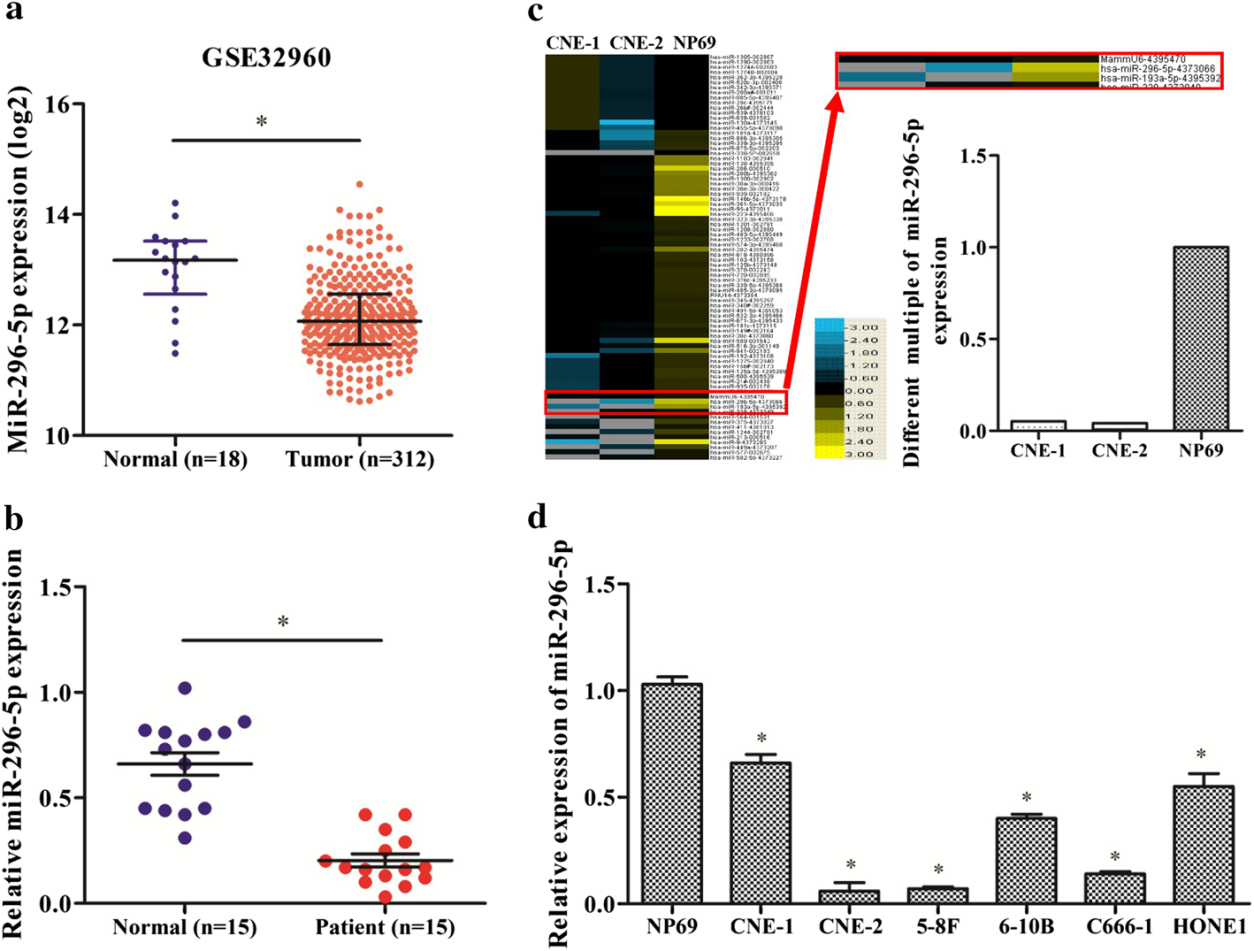
Nasopharyngeal carcinoma (NPC) cells show lower expression of miR-296-5p. **a** The expression levels of miR-296-5p were lower in the NPC datasets from GSE32960. **b** An miRNA microarray analysis showed that miR-296-5p is differentially expressed between NPC and NP69 cells. **c** The miR-296-5p expression level was lower in NPC tissue samples compared with its level in paired normal nasopharyngeal tissue. **d** Real-time PCR confirmed that the expression level of miR-296-5p in NPC cell lines was significantly lower than in NP69 cells. *p < 0.05

The analysis of NPC microarray datasets from GSE32920 showed that miR-296-5p expression is significantly downregulated in NPC tissues compared with normal nasopharyngeal tissues (p < 0.05, Fig. 1a). To further verify the miR-296-5p expression in NPC tissues, real-time PCR was performed using 15 freshly collected NPC tissue samples and paired adjacent normal tissue samples. MiR-296-5p levels were also obviously lower in NPC tissues than in adjacent normal nasopharyngeal tissues (p < 0.05, Fig. 1b). This is consistent with our miRNA microarray assay results for NPC and NP69 cells (Fig. 1c).

The analysis of the correlation between miR-296-5p expression levels and the clinical features of NPC patients showed that miR-296-5p expression significantly correlated with lymph node metastasis (p = 0.017; Table 1). In addition, lower expression of miR-296-5p was found in the NPC cell lines CNE-1, CNE-2, 5-8F, 6-10B, C666-1 and HONE1, than in NP69, the immortalized nasopharyngeal epithelial cell line (Fig. 1d). Collectively, these results demonstrate that miR-296-5p expression is downregulated in NPC and is inversely correlated with tumor metastasis.

**Table 1:** The association of miR-296-5p expression levels with the clinical characteristics of the NPC patients

Clinical characteristics	Patients (n)	miR-296		p value
		Down-regulation	No down-regulation	
Gender				p = 0.600
Male	10	6	4	
Female	5	4	1	
Age				p = 1.000
< 50	8	5	3	
≥ 50	7	5	2	
Histological subtype				p = 1.000
Undifferentiated non-keratinizing	13	9	4	
Differentiated non-keratinizing	2	1	1	
EBV status				p = 0.333
Positive	14	10	4	
Negative	1	0	1	
TNM stage				p = 0.251
I + II	5	2	3	
III + IV	10	8	2	
Lymphatic metastasis				**p = 0.017**
No	5	1	4	
Yes	10	9	1	

Bold value (p = 0.017) indicates statistical differences

### MiR-296-5p inhibited the migration and invasion of NPC cells in vitro

**Fig. 2:**
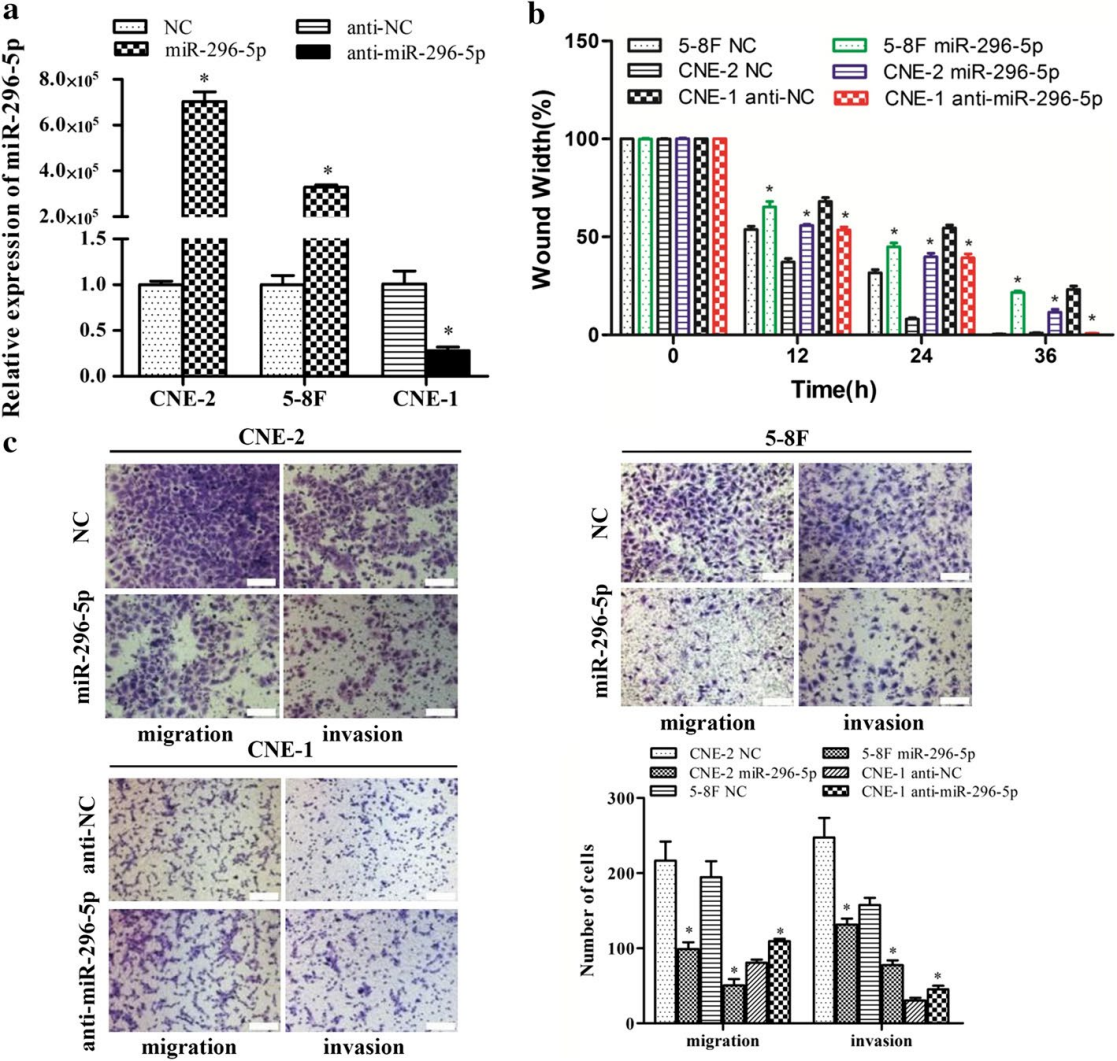
MiR-296-5p inhibits the migration and invasion of NPC cells in vitro. **a** MiR-296-5p expression was determined using real-time PCR. **b** The percentage wound widths for the wound images from wound-healing experiments. **c** Representative photographs of cells from Transwell assays. Scale bar 100 µm, *p < 0.05

MiR-296-5p mimics or inhibitors were transfected into NPC cell lines for functional analyses. Successful transfection was confirmed via real-time PCR. The NPC cells transfected with miR-296-5p mimics showed higher miR-296-5p expression than those transfected with the miR-negative control (Fig. 2a). The miR-296-5p expression in CNE-1 cells treated with the miR-296-5p inhibitor was 28% of that in CNE-1 cells treated with the inhibitor control (Fig. 2a).

The effects of miR-296-5p on the migration and invasion abilities of NPC cells were determined using wound-healing and transwell assays. The upregulation of miR-296-5p significantly suppressed the migration ability of NPC cells (Fig. 2b). Our transwell assay results indicate that the migration and invasion abilities of the CNE-2 and 5-8F cells transfected with miR-296-5p mimics decreased dramatically compared with those of the cells transfected with the miR-negative control (Fig. 2c). Silencing miR-296-5p had the opposite effect in CNE-1 cells (Fig. 2c). These data suggest that miR-296-5p inhibits the invasion and migration abilities of NPC cells.

### MiR-296-5p inhibited the metastasis of NPC cells by regulating EMT

**Fig. 3:**
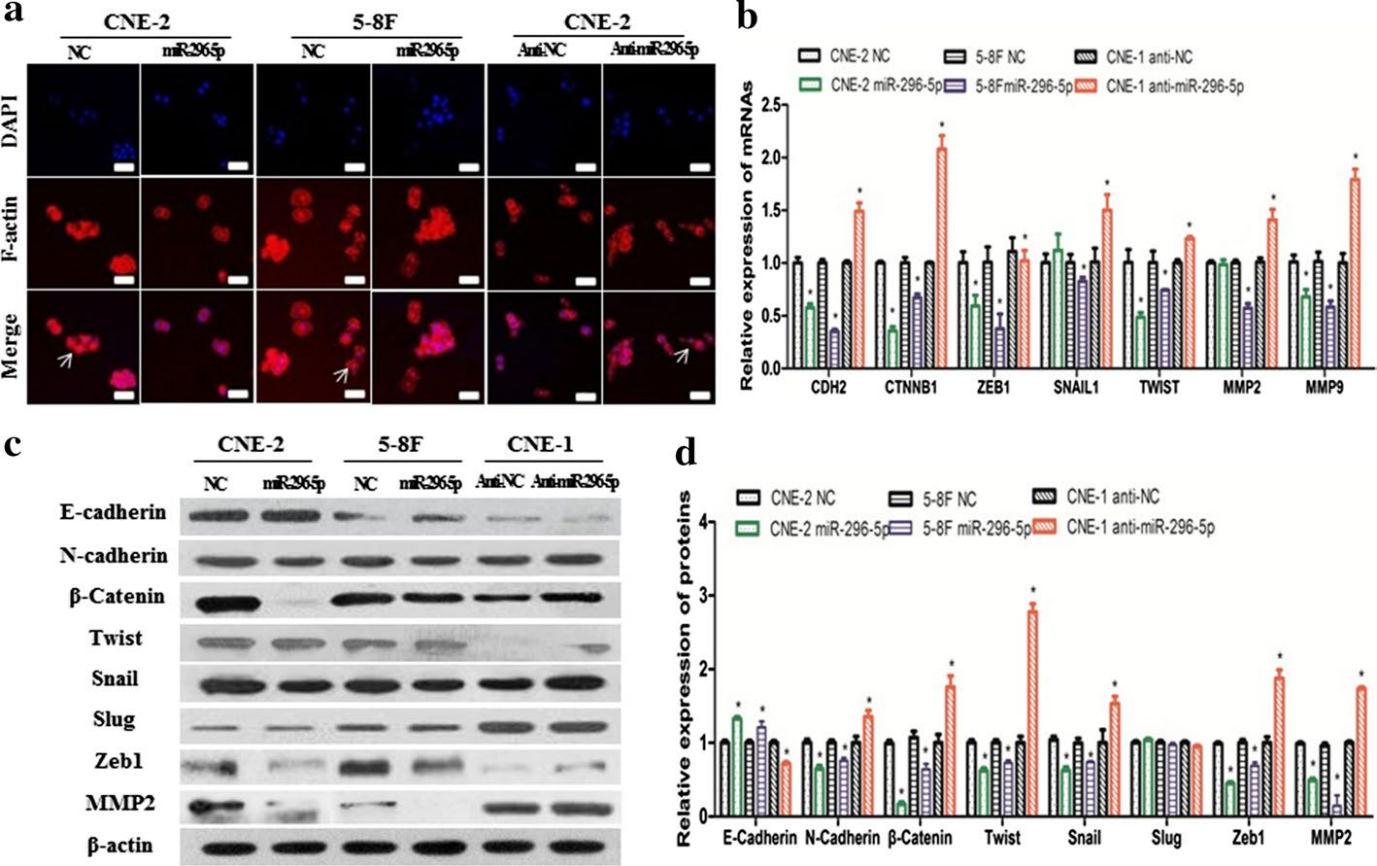
MiR-296-5p inhibits the metastasis of NPC cells by regulating EMT. **a** Immunofluorescence showed miR-296-5p expression affected the morphological changes of NPC cells from the epithelial to mesenchymal type. Scale bar 100 µm. **b** Real-time PCR showed that miR-296-5p expression affected the expression of the EMT-related markers in NPC cells. **c** Western blotting showed miR-296-5p affected the expression of the EMT-related markers in NPC cells. **d** The relative expression levels of the EMT-related proteins with gray-scale analysis. *p < 0.05

EMT plays a critical role in cancer metastasis, so we assessed whether miR-296-5p is involved in its regulation in NPC cells. We established stable CNE-2 and 5-8F cell lines overexpressing miR-296-5p and a stable CNE-1 cell line with silenced miR-296-5p expression. The miR-296-5p-overexpressing NPC cells maintained a typical epithelial morphological phenotype compared with the negative control cells, whereas the miR-296-5p-silenced cells showed the scattered spindle-like morphology of a mesenchymal phenotype (Fig. 3a). Then, we performed real-time PCR and western blot assays to determine the expression of EMT-related markers and transcription factors. The protein and mRNA levels of N-cadherin, β-catenin, Twist, Snail, Slug, Zeb1, and MMP2 were significantly downregulated in the miR-296-5p-overexpressing NPC cells but upregulated in the miR-296-5p-silenced cells (Fig. 3b, c). Conversely, the protein and mRNA levels of E-cadherin, a typical epithelial cell marker, increased in the miR-296-5p-overexpressing cells but decreased in the miR-296-5p-silenced cells. These data suggest that miR-296-5p inhibits the migration and invasion of NPC cells, possibly by hindering EMT.

### TGF-β is the target gene of miR-296-5p

**Fig. 4:**
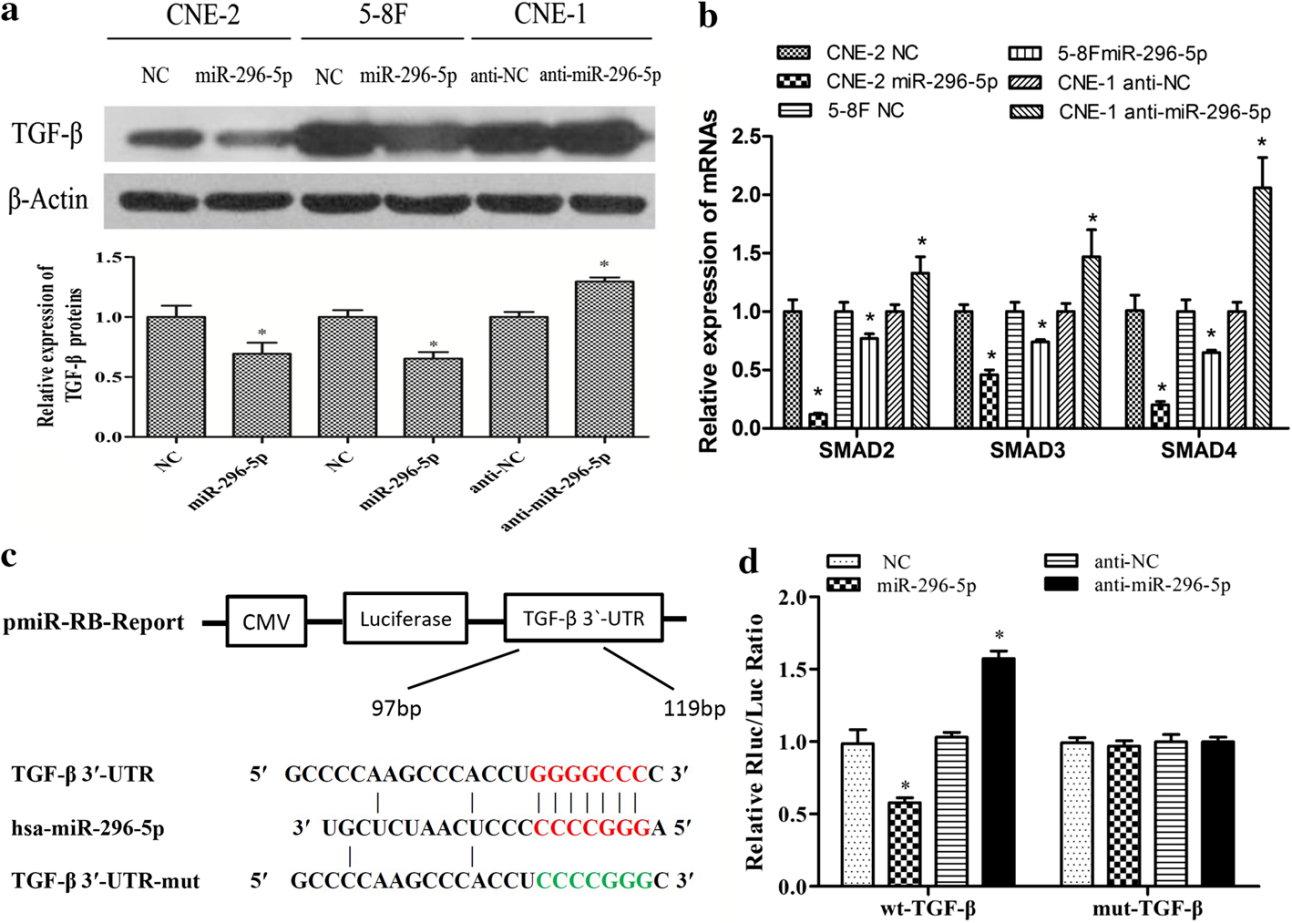
MiR296-5p suppresses TGF-β expression by directly targeting its 3ʹ-UTR. **a** Western blotting showed miR-296-5p expression inversely affected TGF-β expression in NPC cells. **b** Real-time PCR showed that miR-296-5p expression affected the downstream gene expression of TGF-β in NPC cells. **c** The binding sites of miR-296-5p with wild-type or mutant TGF-β in 3ʹ-UTR. **d** Luciferase assays in 293T cells treated with both wild-type or mutant TGF-β vectors along with an miR-296-5p mimic, inhibitor or relevant NC

To identify the mRNA targets of miR-296-5p, we applied the publicly available algorithm TargetScan. TGF-β, a gene that plays an great role in tumor progression by inducing EMT, was identified as a potential target (Additional file 1). Western blot analysis showed that TGF-β expression significantly decreased in the CNE-2 and 5-8F NPC cells transfected with miR-296-5p mimics but significantly increased in the miR-296-5p-silenced CNE-1 cells (Fig. 4a). MiR-296-5p overexpression or downregulation respectively reduced or upregulated TGF-β-mediated SMAD2, SMAD3 and SMAD4 gene expression (Fig. 4b).

Luciferase assays were performed to verify whether or not miR-296-5p-mediated TGF-β downregulation occurs through a miR-296-5p-binding site located in the TGF-β 3ʹ-UTR. Briefly, we constructed pmiR-RB-REPORT luciferase reporter plasmids containing regions of the 3ʹ-UTR of TGF-β and co-transfected them into 293T cells with the miR-296-5p mimics, miR-296-5p inhibitor, or the corresponding negative controls (Fig. 4c). Our results indicate that the miR-296-5p mimics largely decreased the luciferase activity of the TGF-β reporter plasmid, while the miR-296-5p inhibitor elevated the activity of the TGF-β reporter plasmid. However, the miR-296-5p mimics and miR-296-5p inhibitor exerted no obvious effect on the luciferase activity of the mut-TGF-β reporter plasmid (Fig. 4d).

These results support the hypothesis that miR-296-5p negatively regulates TGF-β expression in NPC cells via directly binding to its 3ʹ-UTR.

### TGF-β overexpression rescues the miR-296-5p-induced inhibition of tumor metastasis via EMT in NPC cells

Since miR-296-5p suppressed NPC cell invasion and directly targeted the expression of TGF-β, which is one of the most common factors for EMT induction, we investigated whether the miR-296-5p-mediated inhibition of EMT in NPC cells could be rescued by exogenous TGF-β. We treated CNE-2 and 5-8F cells with miR-296-5p mimics together with exogenous TGF-β and then determined their migration, invasion and EMT phenotypes (Additional file 2).

**Fig. 5:**
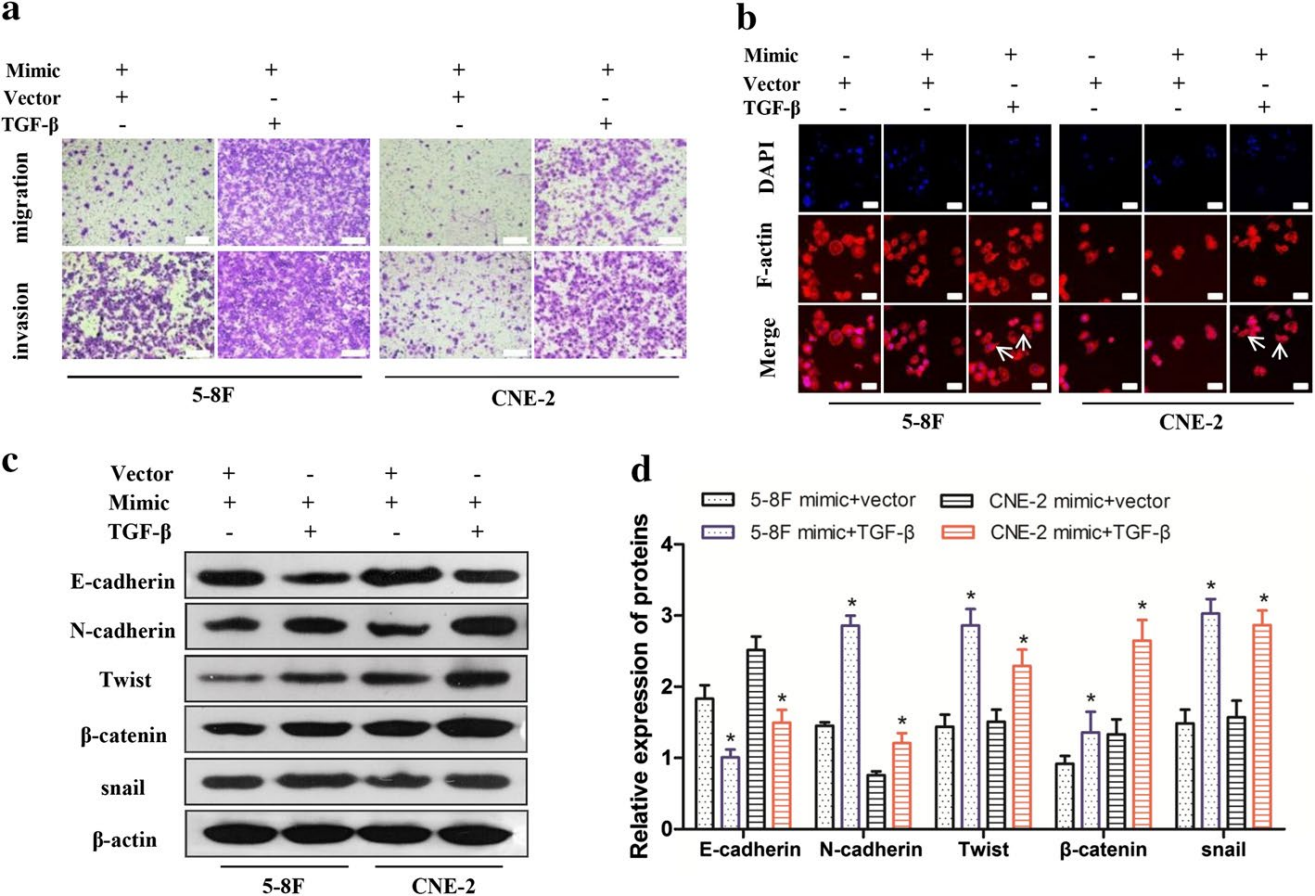
TGF-β overexpression rescues the miR-296-5p-induced inhibition of tumor metastasis in NPC cells via EMT. **a** Transwell assays showed that exogenous TGF-β rescued the miR-296-5p-induced inhibition of metastasis in NPC cells. Scale bar 100 µm. **b** Immunofluorescence showed exogenous TGF-β rescued the miR-296-5p-induced transition to epithelial-like cells. Scale bar 100 µm. **c** Western blotting confirmed exogenous TGF-β rescued the expression of the EMT-related proteins in NPC cells. **d** The relative expression levels of the EMT-related marker gene proteins with gray-scale analysis. *p < 0.05)

In the presence of exogenous TGF-β, miR-296-5p failed to suppress NPC cell migration and invasion (Fig. 5a). Morphologically, exogenous TGF-β was still able to induce a scattered spindle-like mesenchymal phenotype in miR-296-5p-treated NPC cells (Fig. 5b). Consistent with such a phenotypic change, a western blot assay revealed that exogenous TGF-β attenuated miR-296-5p-mediated upregulation of the epithelial marker E-cadherin but robustly reversed the miR-296-5p-mediated downregulation of N-cadherin, β-catenin, Snail, and Twist in NPC cells (Fig. 5c, d). These data strongly support miR-296-5p suppression of cell migration and metastasis through the targeted inhibition of TGF-β-induced EMT in NPC cells.

### Clinical relevance of miR-296-5p with TGF-β in NPC tissues

**Fig. 6:**
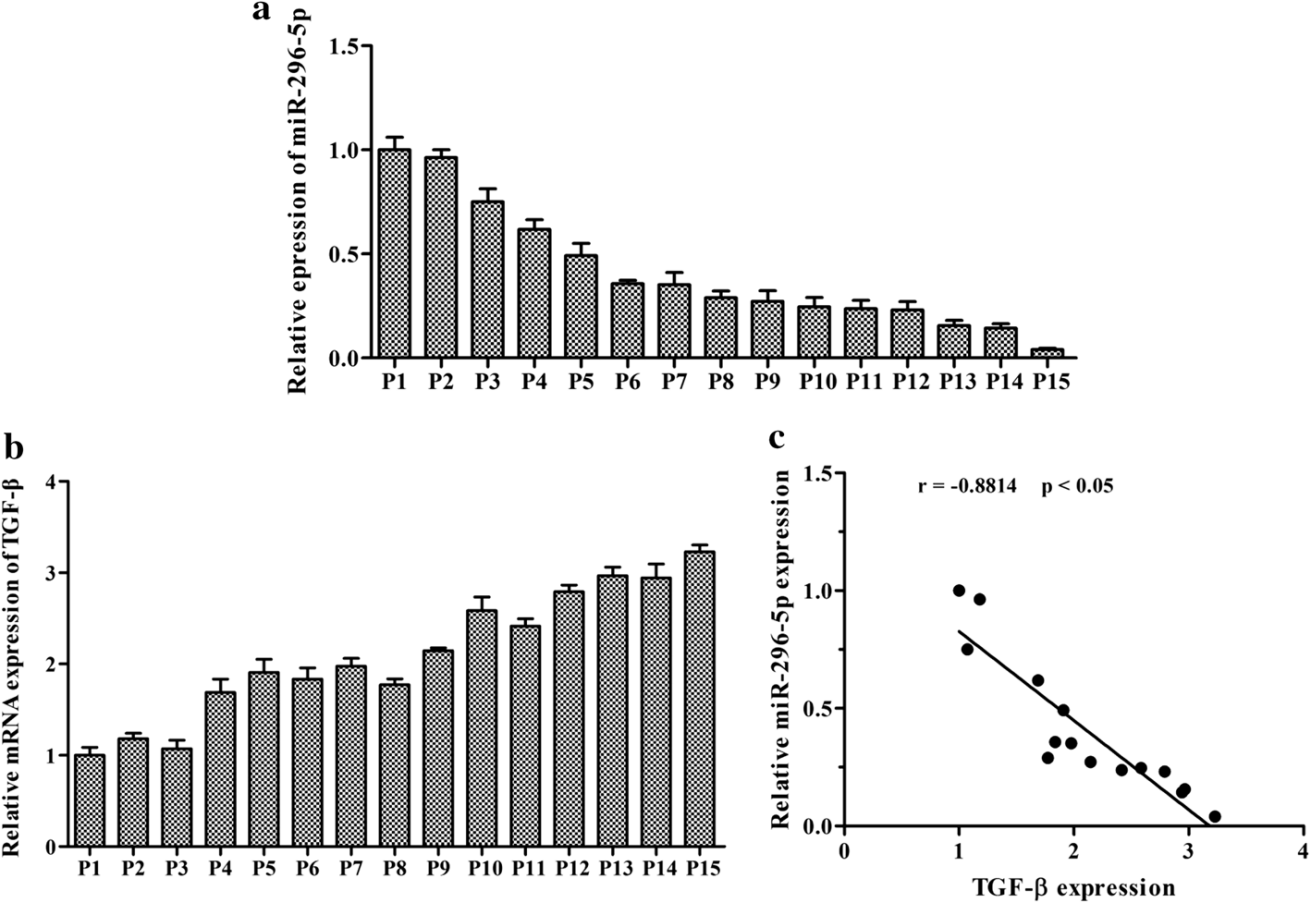
Clinical relevance of miR-296-5p with TGF-β in NPC tissues. **a** MiR-296-5p expression in human NPC tissues. **b** TGF-β expression in human NPC tissues. **c** The correlations between miR-296-5p levels and TGF-β expression in NPC tissues were calculated with Pearson’s correlation tests. *p < 0.05)

To validate the potential clinical relevance of miR-296-5p and TGF-β interaction in NPC, we examined miR-296-5p and TGF-β mRNA expression in 15 NPC tissues using real-time PCR. The tissues were numbered P1 to P15 based on their expression level of miR-296-5p or TGF-β, which were normalized to the corresponding expression levels in P1 tissue. Spearman’s correlation test was performed for the correlation analysis between miR-296-5p and TGF-β expression in the NPC tissues. Our results reveal a significant negative correlation between miR-296-5p and TGF-β expression (r = − 0.8814, p < 0.05) in NPC (Fig. 6). Our findings indicate that TGF-β expression negatively correlates with miR-296-5p levels in NPC tissues.

## Discussion

MiR-296 functions as a potential tumor suppressor in various types of cancer due to its inhibitory effects on tumor cell proliferation and metastasis through direct regulation of its target genes [21–25]. However, little is known about the expression and role of miR-296-5p in NPC.

In this study, decreased expression of miR-296-5p was identified in NPC tissues and cells, which is consistent with its expression profile in pancreatic cancer [26], ovarian cancer [22], thyroid carcinoma [23], hepatocellular carcinoma [25] and colorectal cancer [24]. Importantly, we found that the upregulation of miR-296-5p obviously inhibited the metastasis and invasion of NPC cells. Silencing miR-296-5p had the opposite effect, suggesting that it is closely related to the invasion and metastasis of NPC cells. This further confirmed that it has the same tumor suppressor function in NPC as had been reported for hepatocellular carcinoma [25, 27], ovarian cancer [22], colorectal cancer [28] and cervical cancer [29]. However, miR-296-5p was found to be significantly upregulated in gastric cancer [30] and glioblastoma [31], indicating that it may exert different functions in different types of tissues or organs (Additional file 3, Fig. S1).

EMT, which converts polarized epithelial cells into mesenchymal-like cells, is the primary process involved in tumor cell invasion and metastasis [32, 33]. Our current findings indicate that silencing miR-296-5p expression induces a scattered spindle-like morphology of the mesenchymal cell phenotype in NPC cells. It also significantly downregulated the expression of the epithelial marker E-cadherin, while robustly upregulating the expression of a panel of EMT-regulatory genes, including N-cadherin, β-catenin, Twist, Snail, Slug, Zeb1, and MMP2 in NPC cells. Overexpression of miR-296-5p had the opposite effect on EMT in NPC. These findings support the notion that miR-296-5p negatively regulates EMT in NPC cells.

EMT plays a central role in the complex process of tumor metastasis [34]. TGF-β is a key regulatory factor of EMT: it positively regulates the expression of a panel of transcription factors, including Twist, Snail, Slug, and Zeb1 [35–38], which are all involved in tumorigenesis and metastasis [39]. Our results showed that:

1. TGF-β expression negatively correlates with miR-296-5p levels in NPC tissues.
2. Overexpression of miR-296-5p mimics downregulates TGF-β expression in NPC cells.
3. MiR-296-5p can directly bind to the 3ʹ-UTR of the TGF-β gene.

In addition, the miR-296-5p-mediated inhibition of cell migration/invasion, the EMT phenotype and the downregulation of EMT-regulatory genes was significantly abrogated in the presence of exogenous TGF-β. Our findings support the hypothesis that miR-296-5p is an immediate negative regulator of TGF-β and that it suppresses cell invasion and metastasis by directly inhibiting TGF-β-induced EMT in NPC cells.

This study provides novel insights into the role of miR-296-5p in the regulation of NPC metastasis and invasion. Downregulation of miRNA-296-5p expression in NPC may contribute to the aberrant activation of the TGF-β/SMAD signaling pathways, which results in progressive EMT-driven metastasis in NPC. This elucidation of the mechanisms underlying the interaction between miR-296-5p and the activation of TGF-β signaling pathways will enable us to develop new strategies for the treatment of NPC.

## Supplementary information

**Additional file 1:** Ethical approval.

**Additional file 2:** Original Data.

**Additional file 3: Figure S1.** Representative photographs of cells in wound-healing assays. Scale bar = 100 µm.

## Abbreviations

TGF-β: Transforming growth factor-β
NPC: Nasopharyngeal carcinoma
EMT: Epithelial–mesenchymal transition
miR-296-5p: MicroRNA-296-5p
3ʹ-UTR: 3ʹ Untranslated region
MMP2: Matrix metalloproteinase 2
