# Supplementary material for: MicroRNA-296-5p inhibits cell metastasis and invasion in nasopharyngeal carcinoma by reversing transforming growth factor-β-induced epithelial–mesenchymal transition
Source: Cell Mol Biol Lett. 2020 Nov 3;25:49. doi: 10.1186/s11658-020-00240-x (PMC7640465; doi:10.1186/s11658-020-00240-x)
Supplement: Supplementary file 1 — Additional file 1: Ethical approval. [file 11658_2020_240_MOESM1_ESM.pdf]

伦理审查批件

批件号	PJ2020-027	受理编号	2020-027-01
项目名称	miRNAs 在鼻咽癌中的表达及参与肿瘤发生发展的机制研究		
试验药品名称		临床研究分期	其他
申办方	自筹经费		
合同研究组织			
研究单位	广东医科大学附属医院		
主要研究者	唐旭东、罗海清、李祥勇		
审查方式	□会议审查■简易审查		
审查类别	初始审查	会议审查日期	NA
审查委员	梁政，曾小五		
审查文件	1. 初始审查申请<br>2. 课题研究方案 （版本号： V 2.0, 版本日期：2019 年 03 月 31 日）<br>3. 课题研究方案签字页 （版本号： V 2.0, 版本日期：2019 年 03 月 31 日）<br>4. 知情同意书 （版本号： V 1.1, 版本日期：2019 年 07 月 25 日）<br>5. 招募受试者的材料 （版本号： V2.0, 版本日期：2020 年 04 月 06 日）<br>6. 课题研究计划书<br>7. 课题研究计划书签字页<br>8. 研究团队信息及研究者专业履历（包含研究团队信息表、主要研究者及其他参与研究者简历、资质证明及 GCP 培训证明）<br>9. 机构立项表<br>10. 研究者利益冲突声明		
批准文件	1. 课题研究方案 （版本号： V 2.0, 版本日期：2019 年 03 月 31 日）<br>2. 课题研究方案签字页 （版本号： V 2.0, 版本日期：2019 年 03 月 31 日）<br>3. 知情同意书 （版本号： V 1.1, 版本日期：2019 年 07 月 25 日）<br>4. 招募受试者的材料 （版本号： V2.0, 版本日期：2020 年 04 月 06 日）		

	5.课题研究计划书<br>6.课题研究计划书签字页<br>7.研究团队信息及研究者专业履历（包含研究团队信息表、主要研究者及其他参与研究者简历、资质证明及 GCP 培训证明）<br>8.机构立项表<br>9.研究者利益冲突声明

审查意见

根据卫生部《涉及人的生物医学研究伦理审查办法》（2016）、CFDA《药物临床试验质量管理规范（2003）》、《药物临床试验伦理审查工作指导原则》（2010年）、《医疗器械临床试验质量管理规范（2016）》、《人类遗传资源管理暂行办法（1998）》、《人类遗传资源采集、收集、买卖、出口、出境审批行政许可事项服务指南（2015）》、WMA《赫尔辛基宣言》和 CIOMS 《人体生物医学研究国际道德指南》的伦理原则，经本伦理委员会审查，同意按所批准的临床研究方案、知情同意书、招募材料开展本项研究。

请遵循 GCP 原则、遵循伦理委员会批准的方案开展临床研究，保护受试者的健康与权利。

研究开始前，请申请人完成临床试验注册。符合《人类遗传资源管理暂行办法（1998）》适用范围的研究项目，需要取得遗传办批件，并提交本中心伦理委员会备案后方可启动项目。

研究过程中若变更主要研究者，对临床研究方案、知情同意书、招募材料等的任何修改，请申请人提交修正案审查申请。

发生严重不良事件，请申请人在获知后 24 小时内提交严重不良事件报告到报告伦理委员会。

请按照伦理委员会规定的年度/定期跟踪审查频率，申请人在截止日期前 1 个月提交研究进展报告；申办者应当向组长单位伦理委员会提交各中心研究进展的汇总报告；当出现任何可能显著影响试验进行、或增加受试者危险的情况时，请申请人及时向伦理委员会提交书面报告。

研究纳入了不符合纳入标准或符合排除标准的受试者，符合中止试验规定而未让受试者退出研究，给予错误治疗或剂量，给予方案禁止的合并用药等没有遵从方案开展研究的情况；或可能对受试者的权益/健康以及研究的科学性造成不良影响等违背 GCP 原则的情况，请申办者/监察员/研究者提交违背方案报告。

申请人暂停或提前终止临床研究，请及时提交暂停/终止研究报告。

完成临床研究，请申请人提交研究完成报告。

年度/定期跟踪审查频率	12 个月
下次跟踪审查截止日期	2021 年 04 月 16 日
批件有效期	2020-04-16 至 2021-04-01

伦理委员会 GCP 声明	
我院伦理委员组成及操作方式严格遵循 GCP（包括 ICH-GCP）及相关法律、法规的规定，实施各项操作规程。	
主任委员签字	
伦理委员会	广东医科大学附属医院机构审查伦理委员会（盖章）
日期	2020 年 04 月 16 日
