# Supplementary material for: MicroRNA-296-5p inhibits cell metastasis and invasion in nasopharyngeal carcinoma by reversing transforming growth factor-β-induced epithelial–mesenchymal transition
Source: Cell Mol Biol Lett. 2020 Nov 3;25:49. doi: 10.1186/s11658-020-00240-x (PMC7640465; doi:10.1186/s11658-020-00240-x)

Original data of western blot

Figure 3

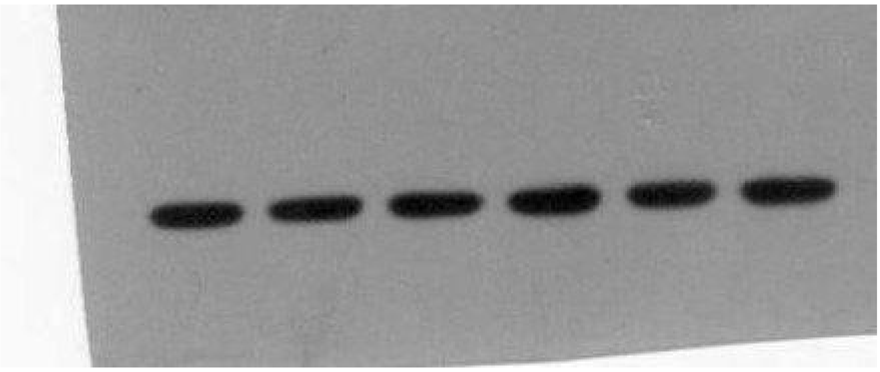

Actin

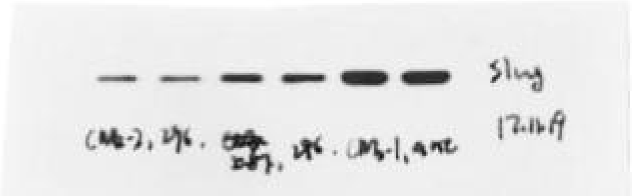

Slug

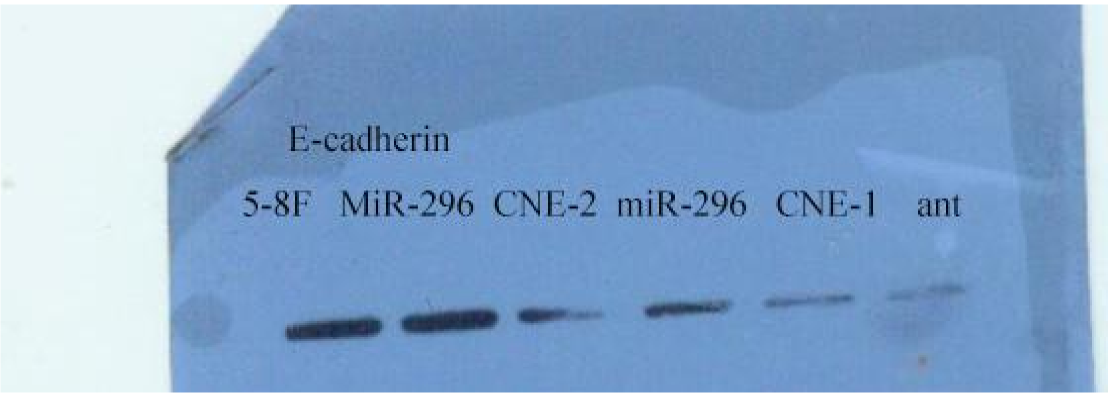

E-Cadherin

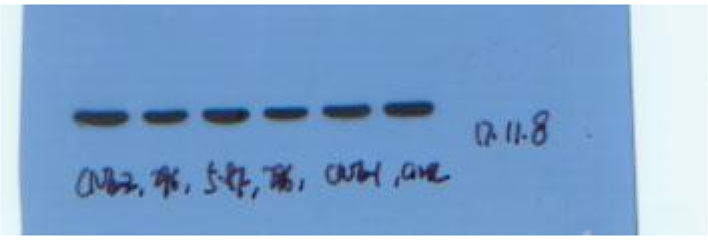

N-Cadherin

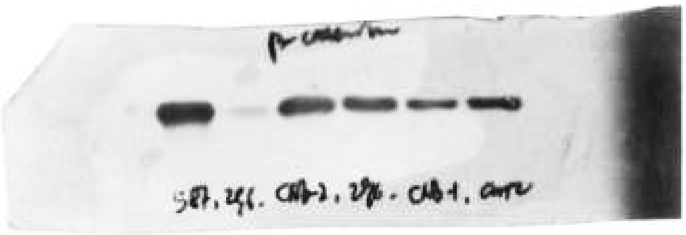

β-catenin

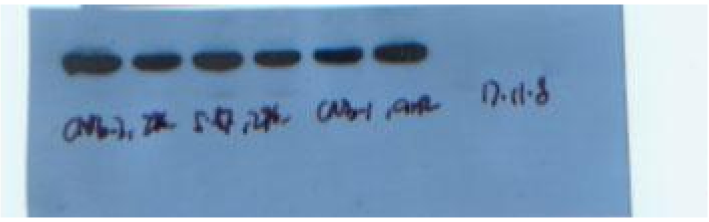

Snail

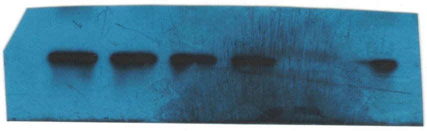

Twist

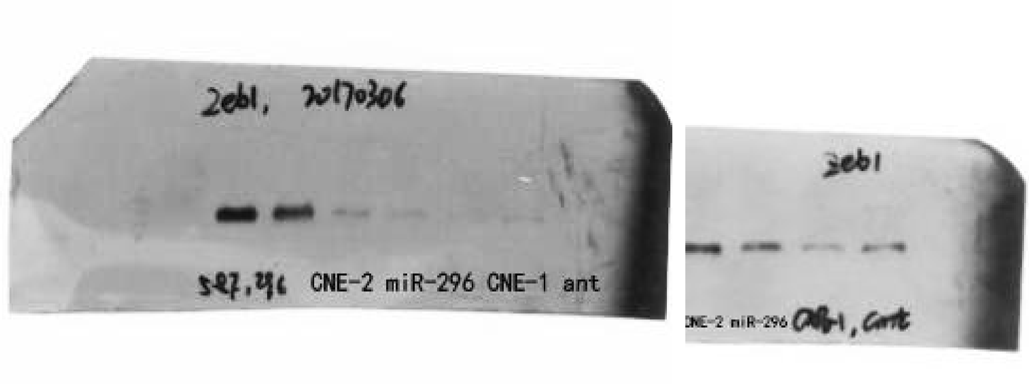

Zeb1

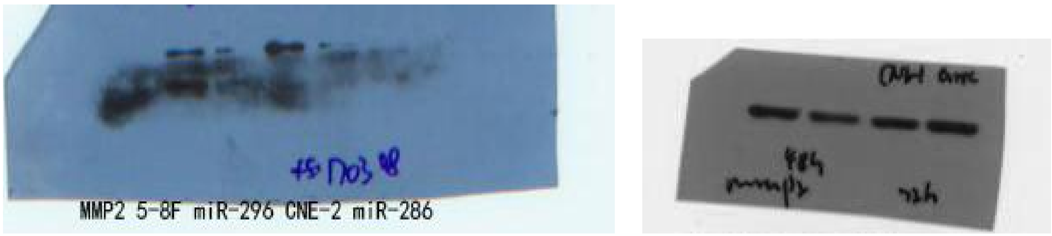

MMP2

Figure 4

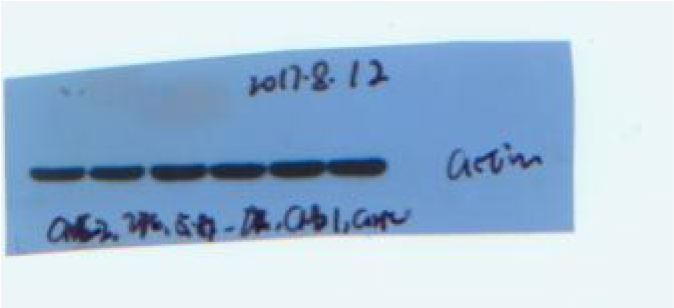

Actin

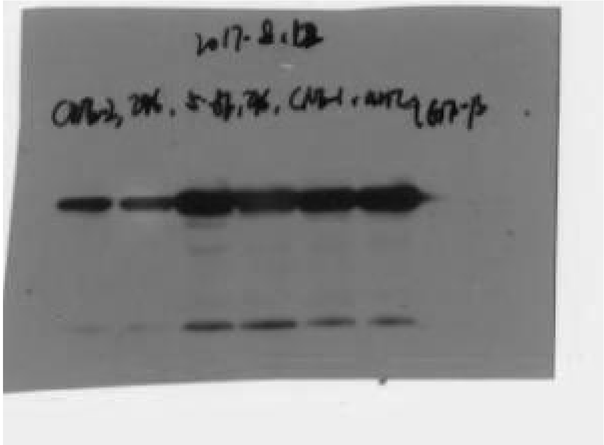

TGF-β

Figure 5

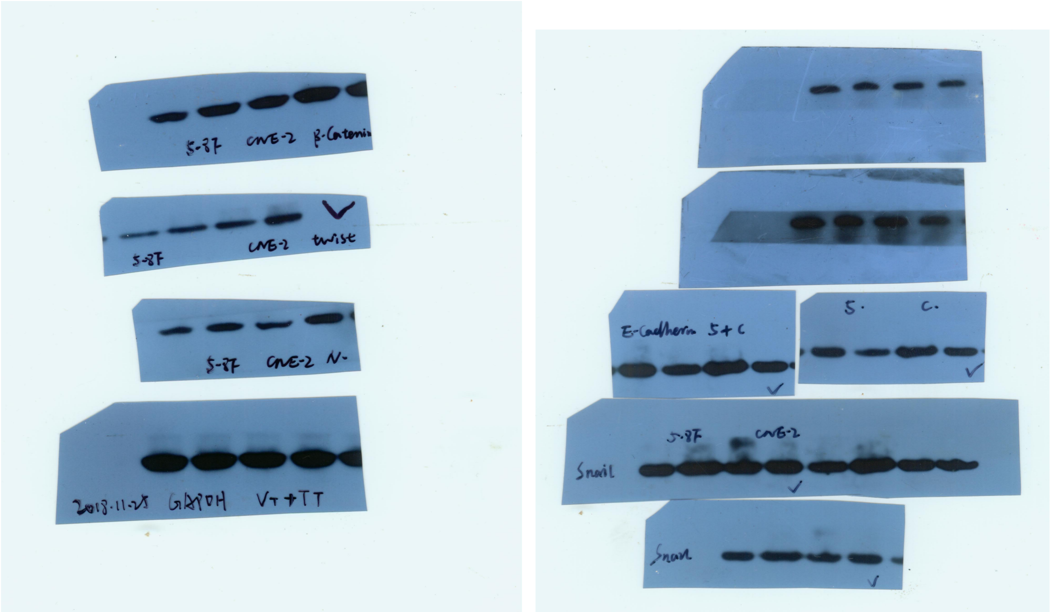

Supplement: Supplementary file 2 — Additional file 2: Original Data. [file 11658_2020_240_MOESM2_ESM.pdf]
