# Supplementary figures and images for: MicroRNA-296-5p inhibits cell metastasis and invasion in nasopharyngeal carcinoma by reversing transforming growth factor-β-induced epithelial–mesenchymal transition
Source: Cell Mol Biol Lett. 2020 Nov 3;25:49. doi: 10.1186/s11658-020-00240-x (PMC7640465; doi:10.1186/s11658-020-00240-x)

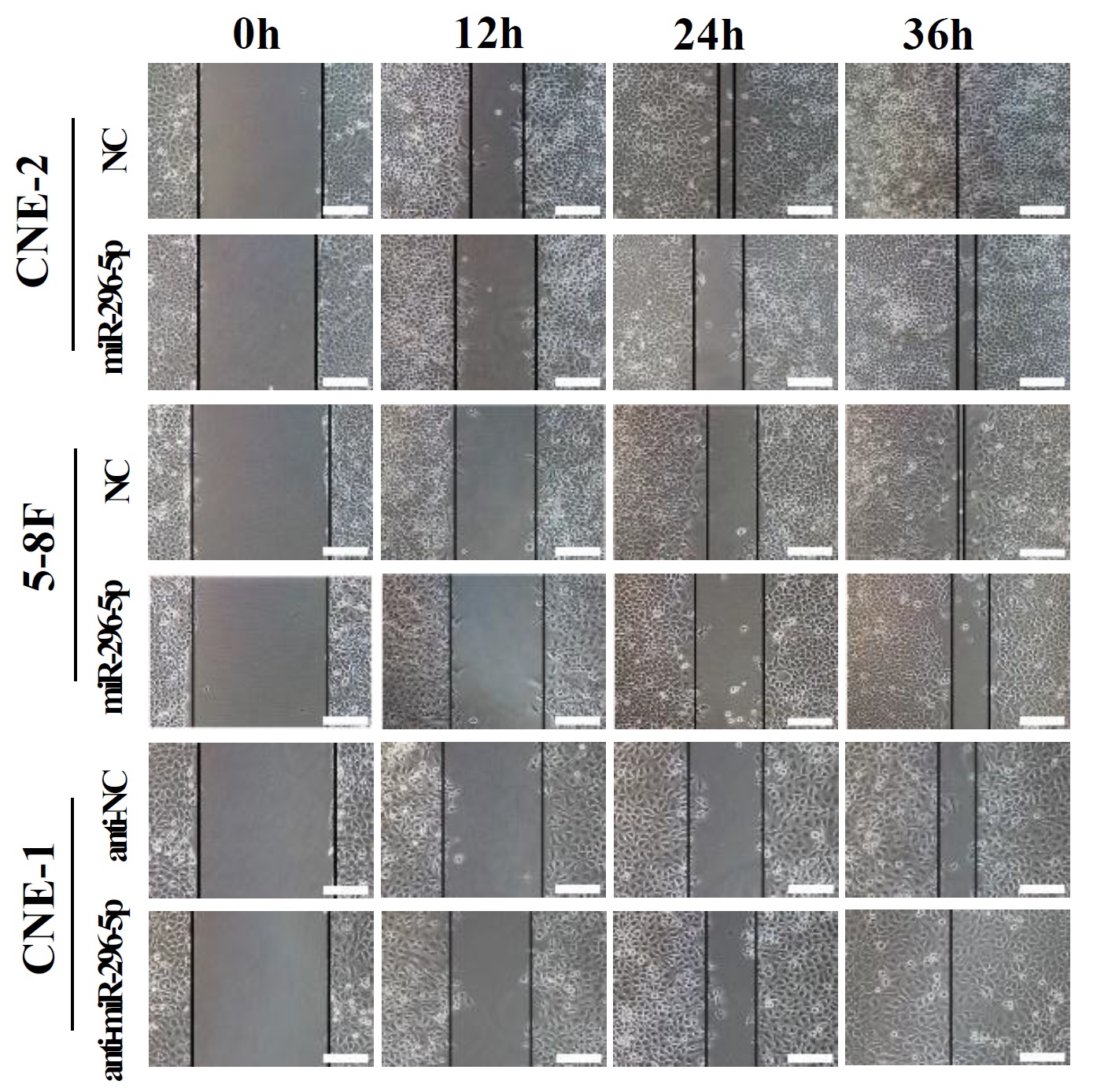

Supplement: Supplementary file 3 — Additional file 3: Figure S1. Representative photographs of cells in wound-healing assays. Scale bar = 100 µm. [file 11658_2020_240_MOESM3_ESM.jpg]
